# What matters to you? An observational field study of patient and care provider expectations for health care relationships

**DOI:** 10.1371/journal.pone.0304854

**Published:** 2024-07-02

**Authors:** Cheryl Rathert, Jessica N. Mittler, Timothy J. Vogus, Yuna S. H. Lee

**Affiliations:** 1 Department of Health Management and Policy, College for Public Health and Social Justice, Saint Louis University, St. Louis, MO, United States of America; 2 Department of Health Administration, College of Health Professions, Virginia Commonwealth University, Richmond, VA, United States of America; 3 Department of Organizational Studies, Owen Graduate School of Management, Vanderbilt University, Nashville, TN, United States of America; 4 Department of Health Policy and Management, Mailman School of Public Health, Columbia University, New York, NY, United States of America; University of KwaZulu-Natal College of Health Sciences, SOUTH AFRICA

## Abstract

Therapeutic connections (TC) between patients and providers are foundational to patient-centered care, which is co-produced between patients and care providers. This necessitates that we understand what patients expect from TCs, the extent to which providers know what patients expect, and what providers expect. The purpose of this study was to examine nine TC dimensions and determine which are most important to patients, which dimensions providers believe are most important to patients, and which are most important to providers. An online survey of patients (n = 388) and care providers (n = 433) was conducted in the USA in March 2021. Respondents rated the extent to which the nine TC dimensions were important to them, followed by open-ended questions to expand upon what matters. The quantitative responses were rank-ordered and rankings were compared across groups. All groups ranked “having the patient’s best interest in mind no matter what” as the top expectation. Patients also ranked “caring commitment” and being “on the same page” as highly important. Providers were relatively accurate in ranking what they believed was most important to patients. Respondents affirmed the TC dimensions in the qualitative results, adding nuance and context, such as patients feeling “heard” and noting providers that go “above and beyond.” Providers ranked dimensions differently for themselves, prioritizing “full presence” and “emotional support” of patients. This study is among the first to examine expectations for TC. TC could play an explanatory role in understanding variation in patient experience ratings and other outcomes.

## Introduction

Understanding patient experiences and patient-centered care has been a major focus among scholars, practitioners, and health care leaders, for several decades [1]. It has been assumed (and empirically demonstrated) that the extent to which patient expectations are met or not influences several important outcomes, such as satisfaction, loyalty, treatment adherence, and even lifestyle choices [1–6]. Recent work has turned attention toward the idea of health care as a service that is co-produced among patients and provider(s) [7–9]. The trend toward patient-centered care, as opposed to provider-centered, exemplifies the concept of co-production in that patients have more voice in their care and providers need to be more attuned to what patients expect and want [9]. Some research has examined patient expectations of health care services and clinical outcomes [2, 5, 6]. Less is known about what patients expect related to patient-provider connections and the extent to which providers know what patients expect. This gap is notable because patient-provider relationships and connections are at the core of service co-production, patient-centered care, and other models of effective care such as the Chronic Care Model [7, 10].

According to Batalden and colleagues, the idea of health care as a co-produced service is based on the premise that services are produced and consumed at the same time [7]. This means that each patient actively participates in co-production to greater or lesser extent, and this participation is built upon relationships. Unfortunately, in most modern health care systems, patient-provider relationships are being degraded rather than strengthened, due to increased administrative burdens and problems with technology, among other issues facing systems worldwide [11, 12]. For example, the National Academy of Medicine reported that in the U.S. the job demands of health care providers have increased substantially without a parallel increase in job resources. Job demands include time pressure, poorly designed workflow, staffing shortages, and work encroaching on personal time, all of which lead to burnout [11]. One defining characteristic of burnout is depersonalization, which for health care providers, can interfere with patient and co-worker relationships. Although differences across countries in health insurance and other system features affect access and equity, it is well recognized that broader macro-level trends (e.g., workforce shortages, financial constraints) threaten to disrupt, depersonalize, and degrade patient-provider relationships across health care systems [13]. Such workplace attributes get in the way of patients and providers connecting and building relationships [14]. When providers connect with patients, they are able to glean much more nuanced information than they can learn from the medical record alone [15]. Thus, achieving a therapeutic connection (TC) with patients seems essential for co-production of high-quality health care.

In order to better understand the co-production of health care, it is important to better understand patient-provider TCs: what patients expect, what providers believe patients expect and want, and what providers expect and want as they participate in co-creating health services. Understanding expectations is important because evidence suggests that patient satisfaction is influenced by the extent to which patient expectations have been met [2, 16], and provider well-being may be related to provider expectations as well. Expectations are defined broadly as the “mental picture” one has about how interactions will unfold [4], or standards used to weigh experiences against [17, 18]. Although some research has examined expectations within patient-provider relationships, much of this has been framed narrowly in terms of communication needs for patients [3, 19–21]. However, we view communication as but a pathway to a TC, not as the connection itself [22, 23]. Further, more information is needed about what providers believe about what patients expect, and what providers expect themselves for connections with patients. In this study we focus on developing a richer, more holistic understanding of relational expectations; what patients and providers expect and believe is important to higher quality relationships in the form of TCs.

The purpose of this study is to closely examine what matters most to patients and care providers in terms of interpersonal connections during health care encounters. Using an observational field study approach, we surveyed both patients with recent health care encounters and clinical care providers (physicians, nurses, and advance practice providers or APPs) within the United States (U.S.). We asked patients to rate the importance of specific TC dimensions (idealized expectations), then asked an open-ended question to further gather information about what matters to them. Similarly, we asked care providers what they believe is important to patients in terms of TCs; and what is important to providers themselves. These questions were followed by open-ended questions to probe more deeply into what matters and why and how it does. The findings of this study should improve our understanding of what matters to patients specifically related to TCs, and the extent to which care providers are aware of patient TC expectations. In turn, this research provides a richer empirical foundation for building sustainable patient-centered care, grounded in TCs.

## Background

### Co-production of health services

At the core of the co-production concept lies the patient-provider relationship and interactions. Accordingly, health care services require patients and providers to work together, to varying degrees, to create the service and its value. Services are intangible and are produced and consumed at the same time [7]. Given that patients are increasingly expected to play an active role in their care, through adherence, self-care, and self-management, it is imperative that they have strong connections with providers and health systems. Batalden and colleagues viewed this core as the center of a set of concentric circles that includes varying levels of coproduction, and different services nested within the health care system and society writ large, which all hinder or support the core [7]. Similarly, after decades of research using Wagner and colleagues’ Chronic Care Model to identify effective practices for chronic disease management, the model was revised in 2019 to highlight that the core involves “productive interactions” between “informed, activated patients” and a “prepared, proactive practice team” [10, 24]. We submit that the foundation of productive interactions is the TC.

Thus, in order to improve delivery of patient-centered care, more attention must be paid to the relational aspects of care, and particularly, how patients want that care delivered interpersonally. Batalden and colleagues referred to this paradigm shift as moving from a medicine-as-product to a “service-making logic” that increases value [7, 25]. At the same time, the broader management literature is paying more attention to the relational elements of job characteristics for workers in general, and for health care providers in particular [9, 26, 27]. Stronger patient-provider connections may serve to offer providers nuanced, contextual patient information that can help achieve higher quality in a setting that is suffused with ambiguity and complexity [9, 15]. Further, stronger TCs may increase work meaningfulness for providers, which could help mitigate burnout [15].

### Patient-provider relationships and therapeutic connections

Patient–care provider relationships have been studied for decades and are considered the foundation for high quality care. Much of the research on patient–provider relationships emphasizes patient–provider communication [21, 28, 29]. It is often through communication that relationships emerge and connections get stronger or weaker [21, 29, 30]. A related body of research has focused on patient–centeredness of care [31–34]. Patient–centered care assumes development of a partnership between patients and their providers, where patients are truly at the center of all decisions, not only in their own care but in health systems and communities [35]. This view posits that not only are decisions made with the patient’s input, but also providers and systems must understand and respect each patient’s unique needs and preferences [32]. In their unpacking of the concept of patient–centered care, Mead and Bower put forth the idea that a “therapeutic alliance” is essential in a health care relationship in order to achieve the best patient care [31]. A review of the first 15 years of research on patient-centered care found that most studies assumed patient–centered care occurred in a context of therapeutic alliance or connection, but few studies in the health care literature had ever conceptualized or examined TC [36].

In parallel with the growing emphasis on patient–centered care, practitioners, policymakers, and scholars observed that health care providers were experiencing growing levels of stress and burnout [11, 14]. According to the National Academy of Medicine, a key factor adding to stress and burnout is the inability of providers to therapeutically connect with patients [11]. Stress for providers due to lack of connection has increased since the COVID-19 pandemic and increased use of telehealth visits [37]. Further, a recent systematic review found that interventions designed to improve the patient–care provider relationship not only improve outcomes for patients, but also providers [28].

The present study contributes to this literature by taking a nuanced approach for better understanding of which dimensions of TC are most important to patients and providers and underpin patient-centered care. TC has been defined as, “an empathic interaction in which the provider is fully present and primed to understand the patient, and the patient feels known, heard, and understood by the provider” [23]. A TC also implies that the patient is fully present and primed to connect with the provider. As noted above, TC extends research on communication because stronger relationships are conceptualized as an outcome of communication [21, 29]. It adds to the patient–centered care literature by specifying and unpacking the context (TC) in which patient–centered care occurs [31].

**Fig 1:**
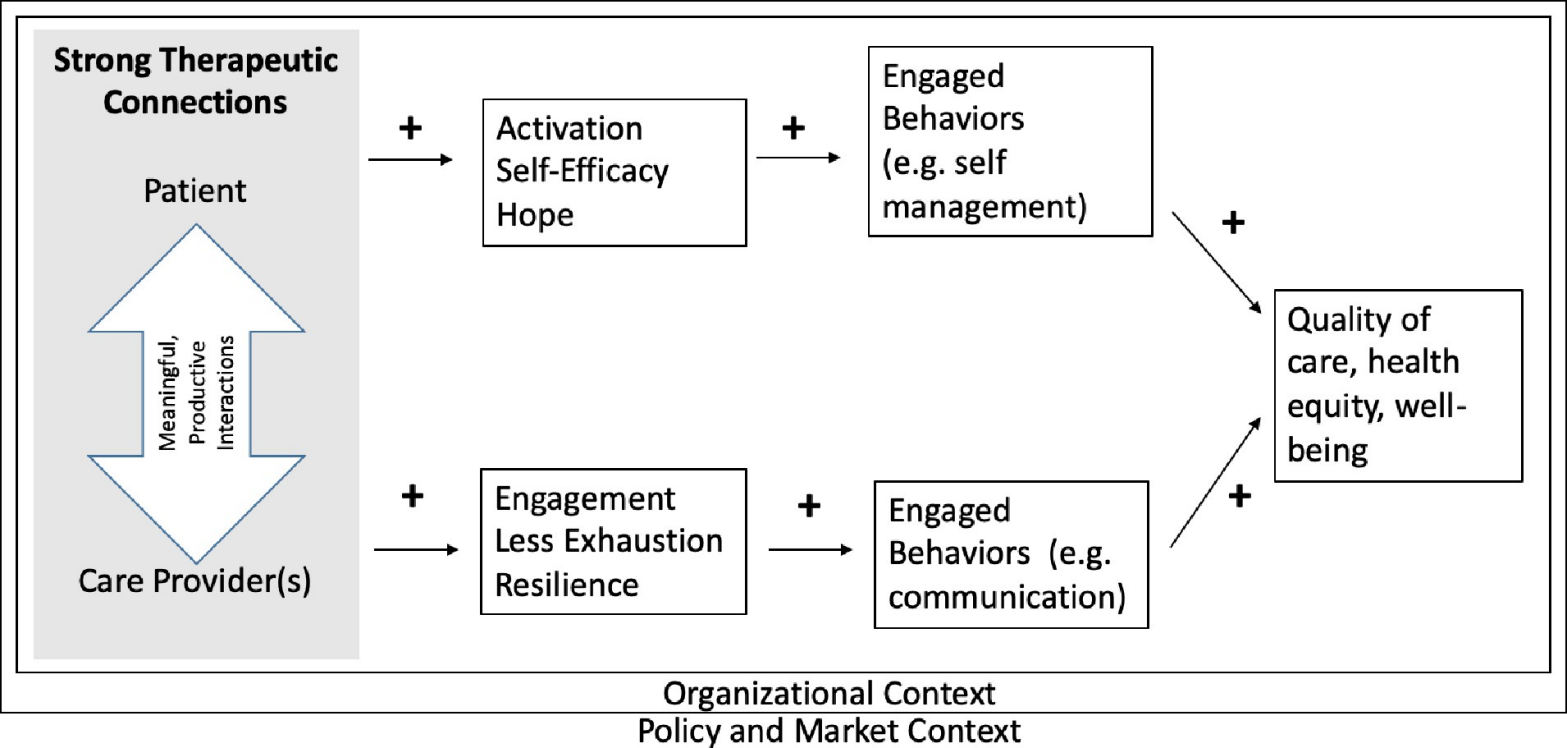
Overarching conceptual model linking patient–provider therapeutic connections and health and well-being outcomes.

Similar to the Chronic Care Model [10] and concepts around co-production [7, 9], we propose that TCs form the core of the patient-provider relationship and facilitate productive interactions, positive patient health and well-being, and positive provider health and well-being; this should increase engagement and communication, and ultimately be related to broader health and well-being. Although we do not test our full model in this study, we keep the full model in mind as we unpack and conceptualize what is important in the TC. Our over-arching conceptual model of how TCs may influence broader outcomes is depicted in Fig 1.

The unpacking of the TC construct has only recently been attempted; yet better understanding of its components may increase understanding of patient expectations and how to meet them. Conceptual work has identified nine TC dimensions from the patient’s perspective Dimensions and their definitions are shown in Table 1. The dimensions were developed following a systematic review of the literature on concepts related to “therapeutic connection” “therapeutic alliance” “patient–provider relationships” and related concepts. [23]. This review also examined measures of the TC concept or its dimensions. At the same time, the research team conducted focus groups with patients to get their perceptions of how relevant and how important various dimensions were for them, and a similar study was conducted with subject matter experts, including academics, clinicians, and health care leaders. The goal of this multi-faceted approach was to identify the most important concepts and dimensions that describe patients’ perceptions of connections. The researchers also captured the words patients used when describing things that matter to them. (See Rathert, Mittler, and Lee [23] for a detailed discussion of the development of the TC concept.) After the nine dimensions were identified, the research team operationalized each dimension by writing one item that seemed to most exemplify that dimension (e.g., items in Table 3). The research team agreed that operationalizing the dimensions in this way would be more meaningful to patients than would presenting them with academic definitions of each dimension; much of the wording of the items included words that patients used in the focus groups (e.g., “we’re on the same page”). This approach differs from past work that sought to identify shared dimensions of therapeutic patient-provider relationships, but primarily through the lens of the provider only [38].

**Table 1:** Therapeutic connection domain definitions.

Dimension	Definition
Shared mind	Mutual understanding of the patient’s values, preferences, and goals; provider understands the “whole self” of the patient; provider is culturally competent
Shared power	Shared power is dynamic; providers are willing to defer to patients and other providers, and patients are willing to defer to providers
Shared deliberation	Shared discussion, negotiation, decision-making about the patient’s diagnosis and treatment
Caring	An interpersonal process involving an emotional commitment to, and concern from provider to patient; empathy
Full presence	Full presence requires total focus of the provider on the patient, without interruptions and distractions
Trust	The patient is vulnerable and believes the provider has their best interests at heart.
Respect	Honoring the humanity of the other regardless of their levels agreement.
Bond	Feelings of attachment to one another, and feelings of commitment to the relationship.
Emotional support	Unconditional compassion: empathy plus action; taking action to support patients’ fears and anxieties

Source: Rathert and colleagues [23] for more information.

### Expectations for TCs

Patient experiences of various health care services have been studied extensively, but less is known about patient expectations for care, especially expectations for patient-provider connections. Research has shown that patient evaluations of their care are at least partially related to the expectations they have for their care [2, 16]. Further, it is unclear the extent to which providers know what patients expect, and what providers expect in terms of TCs in order to do their job effectively. As noted above, patient expectations can be defined as the “mental picture” patients have about how interactions will take place, and patients may not always be consciously aware of these expectations [4]. The customer service literature defines service expectations as a standard that customers use to weigh their experiences against [17, 18]. Patients may have a specific agenda they expect to address during a care encounter, and some expectations on their agenda may be about things other than their current medical condition [5]. One large longitudinal study of unmet patient expectations found that when patients had one or more expectations that were not met, they were less satisfied, and their providers perceived the encounter as “more demanding” and were less satisfied with the visit as well [2]. When providers know what patients expect, they are more likely to be able to support and satisfy expectations that are justified and have discussions about expectations that are not realistic [2].

Although patients may have specific expectations about systems, encounters, or treatments [2, 6, 39], they may have more general expectations about connecting with providers. The customer service literature has focused extensively on customer expectations of service [18, 40]. These models posit varying expectation levels: *ideal* standards are those the customer believes they can and should experience; *predictive* (experience-based) expectations are those customers believe are likely based on previous experiences (i.e., possibly less than ideal); and *adequate* expectations are the lowest level of service customers will tolerate [18]. Ideal expectations remain relatively stable, whereas expectations of what is adequate tend to be more variable [17]. Complicating the ability to understand patient expectations is the finding that customers may use more than one standard at the same time, and/or have different expectations for different dimensions of a service [17, 41]. Further, there is a “zone of tolerance,” which is the gap between the experiences customers expect ideally, and the minimum they will tolerate to consider service adequate [17, 18, 42, 43]. The zone of tolerance varies depending on situational factors, such as the importance of the situation, and there are likely ranges in the zone of tolerance for different dimensions of the service encounter [17]. Because of these nuances, patients could have detailed, encounter-specific expectations based on their individualized reason for seeking care, and at the same time hold broader, more general expectations about relationships and TCs.

In this study, we aim to explore and examine more generalized, idealized expectations of patient-care provider TCs from three different perspectives: (1) what is important to patients; (2) what care providers believe is important to patients; and (3) what is important to care providers. To do this we surveyed patients and care providers and asked them to rate examples of experiences based on the nine empirically established TC dimensions shown in Table 1. Because this was an exploratory study, we aimed to answer the following research questions:

- RQ 1: Which TC dimensions are most important to patients?
- RQ2: Which TC dimensions do care providers understand to be most important to patients?
- RQ3: Which TC dimensions are most important to care providers (physicians and nurses)?

## Method

### Study design

In order to examine our research questions, we conducted an observational field study consisting of cross-sectional, online surveys of both patients and care providers, all of whom were located within the U.S. Any respondent located in the U.S. was eligible for the study. It is notable that unlike many countries, the U.S. does not have universal health care coverage, and this results in health care access inequities. Despite the critical importance of this difference in access, it is important to note that our study focused on patient expectations of TCs for patients who were able to access a health encounter and did so in the previous year. A market research firm was retained to distribute the surveys from its large sample frame of individuals from across the U.S. Respondents in each group who completed surveys were entered into a raffle for cash prizes at the end of the survey period. The authors had no access to participant identification information. The study was approved as exempt from review by the Saint Louis University Institutional Review Board.

### Participants

Patients and providers were recruited by email from March 4–17, 2021, from the research firm’s databases. Although anyone who lived in the U.S. was eligible, most respondents were located in the Northeastern or Midwestern sections of the country. Our targeted sample size was 325 for each survey. Targets were determined based on established practices for recruiting survey study representative samples [44, 45]. These targets were based on a medium effect size, a 3% margin of error, with a 95% confidence level [44]. Further, the targeted sample size was consistent with general power recommendations for correlational studies [46]. The patient sample frame included all individuals in the research firm’s general database; all individuals received an invitation email, which included a letter of consent. If individuals were interested in participating and were over the age of 18 years, they were redirected to the survey and answered the following question: “In the past year, have you had at least one visit with a health care provider for yourself, not a child or other family member? This visit could include either an in-person or telehealth (video) visit.” Those who answered “no” were thanked and exited from the survey. Those who answered “yes” continued the survey. A stratified sampling approach was utilized for the patient sample in order to recruit approximately equal numbers of patients with and without at least one chronic condition, therefore, after indicating they had had a health care visit in the previous year, respondents were asked, “In the past year, have you had a long-term medical condition that required regular medical monitoring or treatment?” Those who answered “yes” were included in the chronic condition category, and those who answered “no” were included in the non-chronic condition category. A minimum quota was set at 150 respondents per group. The survey was closed after 388 patients met inclusion criteria.

The care provider sample frame included physicians and nurses from across the U.S. who were known to the research firm to be physicians and nurses. Similarly, for the provider survey, a stratified sampling approach was used to obtain approximately equal numbers of physicians and nurses. Providers were required to be 18 years or older, employed full time, and to currently spend at least 50% of their time in direct patient care. Because the survey was focused on experiences of TCs with patients, certain physicians and settings were excluded: pediatrics and behavioral health. Providers who worked primarily in pediatrics were excluded because pediatric care in the U.S. typically includes involvement with family members, and the intention of our study was to examine direct interactions between patients and care providers only. We excluded providers that work primarily in behavioral medicine because the concept of TC is similar to “therapeutic alliance,” which is a specific psychotherapeutic process [47] specifically directed at building connections and alliances over time. Thus, including providers using the more specific psychotherapeutic processes would be qualitatively different from the typical patient experience and expectations about TCs with care providers (and providers’ expectations and preferences regarding TCs with patients). The survey closed after 433 providers met inclusion criteria. The majority of care provider respondents were registered nurses (42%). This was followed by physician specialists (25%), nurse practitioners (14%), primary care physicians (8%) physician assistants (4%), and licensed practical nurses (2%). The remaining provider types captured on the survey were: emergency physician (2%), hospitalist physician (2%), other type of physician (1%), and other type of nurse (1%).

### Measures

As noted above, surveys were developed based on previous systematic reviews and qualitative research [23]. The TC constructs and wording of items had been tested in two patient focus groups (n = 22 patients) and with one survey of subject matter experts (n = 24 experts). Wording of survey items utilized patients’ own words from the focus groups. In the interest of patient-centeredness, items were developed first for patients, from the patient’s perspective. One item was developed to exemplify each of the nine dimensions (Table 1). The instructions asked patients to think “what makes a good patient-provider relationship, what matters to you as a patient? When we say “provider” that means your **IDEAL** doctor, nurse, or other type of health care provider.” They were then asked to rate nine items on a 0–10 scale where 0 = Not at all important and 10 = Essential. Example items were, “My provider has my best interests in mind, no matter what” (trust) and “My provider honors me even if he or she disagrees with me on important matters” (respect). Following this set of questions, patients were asked the following open-ended question: “Is there anything else you would like us to know about your relationship with your provider?” This question was intentionally vague in order to provide the opportunity for respondents to reveal things that are important that were not mentioned on the survey.

After the patient items were written and vetted, two items were written for care providers that mirrored each of the nine patient items: one item asked the extent to which providers believed their patients found each item important; and a second asked providers how important each item was to them. Instructions were: “For the following questions, please think about what matters in patient-provider relationships from your patients’ perspectives. What do you believe matters to your patients?” As with the patient questions, providers rated each item on a 0–10 scale where 0 = Not at all important and 10 = Essential. Example items include: “The patient believes their provider has their best interests in mind, no matter what” and “The patient believes their provider honors them even if the provider disagrees with them on important matters.” This set of questions was followed by an open-ended question that asked, “Are there other key factors in the patient-provider relationship that you believe are important to patients?”

The second set of provider questions was similar, but items were worded to ask what was important to the providers themselves. Again, one question was developed for each of the nine dimensions and mirrored the patient items, with a response scale of 0–10 where 0 = Not at all important and 10 = Essential. Example items include: “How important is it to have the patient’s best interests in mind, no matter what?” and “How important is it to honor the patient even if we disagree on important matters?” This set of questions was followed by two open-ended questions: “If you could, what would you change about your relationship or interactions with your patients?” and “What is one thing you would change about your work environment that would help you have your ideal patient–provider relationship?” Survey items for both patient and provider surveys (S1 File).

### Analysis

For each set of questions on both patient and provider surveys, mean scores were computed, and the items were rank-ordered based on the mean scores. Because patients with chronic conditions that received regular monitoring may be more likely to have more frequent health care visits than those without chronic conditions, and therefore may have different expectations, we divided them into two groups (chronic conditions/no chronic conditions) and conducted one-way analysis of variance (ANOVA) to compare their mean ratings for each item (not rank orders). Similarly, we divided provider respondents into three groups that best reflected their job types (nurses, advances practice providers [APPs], physicians), rank-ordered the items based on their scores, and utilized one-way ANOVA to compare their mean ratings (not rank orders) to examine the extent to which importance for each item varied based on job type. (Complete ANOVA results are available by request from the first author.)

For analyzing the qualitative data, we used a framework analysis approach for all questions. Framework analysis can be used when the intention is not necessarily to develop theory, but to generate ideas for policy and practice [48]. Data are coded based on pre-existing phenomena (as opposed to inductively developing theory), and respondent comments are kept as a whole rather than parsing them out. The research team members independently coded samples of comments, beginning with general patient experience domains (e.g., access to care, coordination, etc.), and defining characteristics of the nine TC domains (e.g., shared mind, shared deliberation, etc.). Comments were coded to a domain whether they were positively or negatively related to the domain (e.g., a comment was coded to shared power even if the respondent indicated they did not want to share power). The team then met, discussed ambiguous and new examples and concepts, and revised the coding scheme. This process occurred four times, then we felt the codes were clearly defined enough to code all four questions. (The provider question about the work environment included an additional set of codes.) Although most comments addressed more than one content category, results are reported based on only the primary code assigned. Results are reported for each set of questions separately.

## Results

Respondent characteristics are shown in Table 2. Our patient respondent demographics were very similar to typical U.S. patient respondents in other surveys [49–51] with a couple of exceptions: our respondents were slightly younger and slightly more educated than typical patient experience respondents. Both patient and care provider samples included higher percentages of females. However, this is not surprising, as it is well-established that females are more likely to respond to surveys [52]. Further, over half of our care provider sample was comprised of nurses, and nurses are roughly 90% female in the U.S. and most other countries [53]. Therefore, our respondents were demographically consistent with typical survey respondents in the U.S.

**Table 2:** Respondent demographic characteristics.

Variable	Patients (n = 388)	Nurses (n = 205)	Advance Practice Providers (n = 68)	Physicians (n = 160)
**Age**				
18–24	7%	1%	1%	0%
25–34	15%	17%	24%	2%
35–44	13%	26%	44%	10%
45–54	21%	20%	19%	21%
55–64	19%	21%	7%	33%
65–74	21%	15%	6%	28%
75+	4%	0%	0%	2%
**Sex**				
Female	75%	94%	87%	76%
Male	24%	6%	13%	23%
Non-binary	1%	0%	0%	1%
**Hispanic descent**				
Yes	7%	1%	2%	7%
No	93%	99%	98%	93%
**Race** *				
White	84%	90%	84%	79%
Black	10%	6%	8%	1%
Asian	6%	1%	6%	14%
Other**	5%	3%	1%	6%
**Education**				
High school or less	8%	0%	0%	0%
Some college/ 2 year degree	28%	25%	0%	0%
4-year college degree	20%	61%	2%	0%
Post-graduate degree	34%	14%	98%	100%
**Insurance type**				
Employer	57%	na	na	na
Medicare	24%	na	na	na
Medicaid	5%	na	na	na
Other	12%	na	na	na
None	2%	na	na	na

*Multiple responses were allowed

**Other includes Native Hawaiian/Pacific Islander; Native American/Alaskan Native; all other

For the most part, all groups rated each item relatively high in importance, but this was expected because we intentionally used items we knew to be important to patients based on previous research [23]. Patient expectations are presented first and used as the reference point for the provider findings. The most notable finding was that all groups rated their respective item, “the provider always has the patient’s best interest in mind…” as the most important item. In general, providers were accurate in predicting the rank order of patient expectations, and physicians were especially accurate compared with nurses and APPs. At the same time, providers indicated a relatively different rank order from patients of what was important to them.

### Patient expectations

Means and standard deviations for patient ratings, and the rank-order of items shown from most important (1) to least important (9) are shown in Table 3. Although the ranking of content domains was not substantially different for patients with chronic conditions versus those with no chronic conditions, mean importance ratings were higher for patients with chronic conditions for all items except one, and one-way ANOVA found they were statistically higher for five of the domains (Table 3). As noted, both groups ranked “the provider always has the patient’s best interest in mind…” as the highest. Patients with at least one chronic condition rated being on the same page, the provider being open to their opinions, honoring the patient even if they disagree, the provider offering emotional support when necessary, and feeling committed to their relationship with the provider, as significantly higher than patients with no chronic conditions.

**Table 3:** Patient expectations for connection rankings, means, and standard deviations.

Patient Therapeutic Connection Expectation	Overall Patient Rank, Mean(SD) (N = 388)	At Least One Chronic Condition Rank, Mean(SD) (n = 229)	No Chronic Condition Rank, Mean(SD) (n = 153)
Provider always has patient’s best interests in mind (Trust)	**1** 9.26 (1.26)	**1** 9.24 (1.32)	**1** 9.29 (1.16)
Provider has caring commitment & willing to act on patient’s behalf (Caring)	**2** 9.06 (1.39)	**3** 9.07 (1.45)	**2** 9.04 (1.30)
Patient & Provider "on the same page" re: patient’s illness & experiences (Shared Mind)	**3** 8.99 (1.38)	2** 9.15 (1.32)	**4** 8.75 (1.43)
Provider pays full attention to patient during visit (Full Presence)	**4** 8.89 (1.55)	**5** 8.93 (1.51)	**3** 8.82 (1.61)
Provider is open to patient opinions (Shared Deliberation)	**5** 8.83 (1.50)	4** 9.07 (1.24)	**6** 8.48 (1.77)
Patient has influence on important issues related to their well-being (Shared Power)	**6** 8.79 (1.60)	**6** 8.90 (1.57)	**5** 8.62 (1.65)
Provider honors patient even if they disagree on important matters (Respect)	**7** 8.28 (1.91)	8* 8.45 (1.79)	**7** 8.03 (2.06)
Provider recognizes emotions and provides emotional support (Emotional Support)	**8** 8.17 (1.98)	9** 8.41(1.88)	**9** 7.82 (2.07)
Patient is committed to relationship with provider (Bond)	**9** 8.04 (2.20)	7** 8.47 (1.95)	**8** 7.41 (2.39)

Notes: *p < .05

**p < .01; for each * item patients with at least one chronic illness provided significantly higher importance ratings than patients with no chronic conditions.

### Patient qualitative results

Recall that patients were asked: “Is there anything else you would like us to know about your relationship with your provider?” Forty-one percent (n = 158) of patients responded to this question. Of these, 16% of comments pertained primarily to the technical quality of the provider; 13% indicated valuing some level of bond with their provider; 12% made a statement related to their provider’s level of caring; and 8% mentioned general comfort or rapport with their provider. Analysis revealed some previously unidentified language from patients, i.e., roughly 6% of patients indicated their provider went “above and beyond” in caring for them. In many cases, patients indicated their specific topic then elaborated with characteristics describing or defining the topic. Patient comments representing each of these categories appear in Table 4. We quantified and categorized the qualitative data to help identify themes, but we use the qualitative data to add richness and depth to the quantitative data.

**Table 4:** Example patient comments for categories most frequently mentioned (n = 158).

Patient General Comments about Providers	Category	Number (%) of Comments
“This is the first provider I’ve had for this condition that both really seems to know what he’s doing, and believes me about how I’m feeling.” “He did his best and his best saved my life.” “My primary doc diagnosed my breast cancer even though I had just had a clear mammogram. She is very thorough.”	Technical quality	27 (16%)
“I have become friends with my provider. We are similar and able to talk easily. We consult/discuss my problems and come up with solutions. She welcomes me to text her if I need to. She’s not just a provider, she’s a friend.” “I have been with my provider for almost 20 years and for a large HMO, he is a great doctor. I have stuck with him when he has moved medical offices. He is caring, listens.” “I have had him for years and think he’s great.”	Bond	21 (13%)
“I feel I have the empathy factor in terms of what my body is doing and how to manage it in a way that is respectful of my lifestyle/goals.” “A very kind and compassionate doctor.” “He is the most caring person I have met.”	Caring	19 (12%)
“I have Rheumatoid Arthritis and was pregnant last year. Both of my doctors worked together to make me as comfortable as possible!” “She is very easy to talk to and she eases my anxiety.” “He makes me feel comfortable and understands my life situation.”	*General Comfort/* *Rapport*	*13* *(8%)*
“My medical team is always available. They go above and beyond to ensure I’m cared for quickly and efficiently. But most importantly, they listen to the entire story. I am truly blessed.” “…he went out of his way to treat our problems.” “My provider went above and beyond what I expected and even spoke to administrators personally when I had a financial question.”	“Above & beyond”	10 (6%)

Although most comments indicated positive characteristics that patients value and expect, some mentioned expected characteristics but gave examples where expectations had not been met:

“*After 12 years*, *my provider seems like he still does not know me and how my medical conditions affect my life*. *This has truly led to me not receiving the total care I needed when specialists would look to my primary provider for more information but he is unable to provide information about me as person and my life*.”

Many patients clearly want (expect) strong connections with providers; however, some also indicated they understand how the organization of health care is a barrier:

“*…With the pressure these days of number of visits that providers must make within an X amount of time*, *there is not enough time to really spend with the provider for her to get to know me*. *It is unfortunately that healthcare reimbursements are based on the quantity and not quality of care*.*”*

And, although most comments indicated value for relational aspects of care, there were a few that placed lower priority on the relationships:

“*I don’t have a personal relationship with medical professionals*. *I respect their expertise and knowledge and expect professionalism*, *but a "bedside manner" is less important to me*. *Similarly*, *I want the best service for my vehicle*, *but I am not concerned if my mechanic and I are on the same page emotionally and personally*. *This person has skills and knowledge that I don’t have*, *which is why I seek their service*. *I feel the same towards medical professionals*.*”*

### Provider perceptions of patient expectations

Provider means, standard deviations, and rankings of their beliefs about what is important to their patients in relation to patient expectations are shown in Table 5. In general, providers were relatively accurate in perceiving what patients expect, with only slight differences in rankings within the top, middle, and lowest ranking terciles, with one exception. The exception was the item addressing that the provider has “commitment and is willing to act on the patient’s behalf,” which was ranked as the number two item by patients, but provider beliefs about how important this is to patients was roughly in the middle. Another notable finding was the item, “the provider always has the patient’s best interest in mind…” was ranked second by nurses, but first among APPs and physicians (the provider and patient “being on the same page” was ranked first among nurses.) Overall, physicians were the most accurate in rank ordering, although they rated most items lower in importance to patients than did nurses and APPs. Physicians rated being on the same page and the patient having influence on important health issues as significantly less important to patients than did nurses and APPs.

**Table 5:** Provider beliefs about patient expectation rankings, means, and standard deviations.

Provider Beliefs of Patient Expectations	Overall Patient Rank, Mean(SD) (n = 388)	Nurses Rank, Mean(SD) (n = 205)	APPs Rank, Mean(SD) (n = 68)	Physicians Rank, Mean(SD) (n = 160)
Provider always has patient’s best interests in mind (Trust)	**1** 9.26(1.26)	**2** 9.19(1.44)	**1** 9.41(.98)	**1** 9.33(1.01)
Provider has caring commitment & willing to act on patient’s behalf (Caring)	**2** 9.06(1.39)	**6** 8.69(1.50)	**5** 8.79(1.54)	**4** 8.68(1.23)
Patient & Provider "on the same page" re: patient’s illness & experiences (Shared Mind)	**3** 8.99(1.38)	**1** 9.39(1.10)	**2** 9.32(.87)	2* 9.09(.96)
Provider pays full attention to patient during visit (Full Presence)	**4** 8.89(1.55)	**3** 9.16(1.27)	**3** 9.29(1.20)	**3** 9.09(1.12)
Provider is open to patient opinions (Shared Deliberation)	**5** 8.83(1.50)	**5** 8.78(1.39)	**4** 9.09(1.22)	**5** 8.74(1.29)
Patient has influence on important issues related to their well-being (Shared Power)	**6** 8.79(1.60)	**4** 8.83(1.33)	**7** 8.76(1.22)	7* 8.41(1.58)
Provider honors patient even if they disagree on important matters (Respect)	**7** 8.28(1.91)	**7** 8.53(1.66)	**6** 8.78(1.35)	**6** 8.41(1.47)
Provider recognizes emotions and provides emotional support (Emotional Support)	**8** 8.17(1.98)	**8** 8.39(1.62)	**8** 8.74(1.30)	**8** 8.37(1.50)
Patient is committed to relationship with provider (Bond)	**9** 8.04(2.20)	**9** 8.07(1.84)	**9** 8.19(1.59)	**9** 8.14(1.79)

Notes: *p < .05; for each * item physicians had significantly lower ratings than the other two groups; APP = advance practice provider (physician assistant or nurse practitioner).

### Provider perceptions of patient expectation qualitative results

For the provider survey question, “Are there other key factors in the patient-provider relationship that you believe are important to patients?” we received 151 usable respondent comments (35%). Providers converged on topic content more than patients did. Notably, 26% of respondents stated that trust is important to patients; another 13% mentioned general shared deliberation, such as collaboration and communication. An additional 13% indicated that listening, specifically, was the most important factor patients considered. Although listening could be considered part of shared deliberation, much of this content focused on patients being “heard,” so we organized these comments in a separate category. Another category with a relatively large number of comments was shared mind (11%), and specifically, many of these comments indicated that it was important for patients to feel providers understood demographic factors on which patients might feel marginalized, such as, race, culture, LGBTQ status, etc. Table 6 shows these categories and example comments for each.

**Table 6:** Example comments for provider beliefs about patient expectations (n = 151).

Provider Comment	Category	Number (%) of Comments
“Trust is important. Trusting that the provider is making good decisions, seeking advice or referring to another specialist if uncertain, and being knowledgeable about new technologies and medications.” “Patient truly believes provider has full commitment to giving them most efficient and safest treatment for their condition.” “Trust, patient needs to trust that physician will be open and honest with them about situation”	Trust	40 (26%)
“Many patients comment that providers don’t talk with them, they talk to them.” “Mutually agreeing on treatment plans.” “That the provider uses terms the patient can understand discussing their health conditions. The patient doesn’t want to be ‘talked down to.’”	Shared Deliberation	19 (13%)
“Some days you just sit with them and listen for 5–10 minutes and it means the world to them.” “Patients feeling they are heard…” “Patients want the providers to listen to them.”	Listening	20 (13%)
“Understand their living situation and challenges faced.” “A need to be felt understood by their provider.” “Patient knows that the provider will honor their culture, sexual orientation, gender identity and religion.” “Cultural affinity: race, age, religion, culture, social class are factors often not addressed.”	Shared Mind	14 (11%)

### Provider expectations

Provider expectations means, standard deviations, and rankings in relation to patient expectations are reported in Table 7. As noted, the domain “the provider always has the patient’s best interest in mind…” was ranked at the top for each group, which is consistent with the patient rankings. While many of the provider domains were ranked similarly to how patients ranked the domains, there were some notable differences. First, each provider group ranked having a “caring commitment and willingness to act on the patient’s behalf” as ninth, while patients ranked this domain as second in importance. All provider groups ranked paying full attention to the patient during the visit as second most important, whereas patients ranked this as fourth. In addition, providers ranked recognizing patient emotions and providing emotional support as third (physicians ranked this as fourth, but there was a tie for their second ranked item), whereas patients ranked this domain as eighth.

**Table 7:** Provider expectations for patient connections rankings, means, and standard deviations.

Provider Expectations for Connections	Overall Patient Rank, Mean(SD) (n = 388)	Nurses Rank, Mean(SD) (n = 205)	APPs Rank, Mean(SD) (n = 68)	Physicians Rank, Mean(SD) (n = 160)
Provider always has patient’s best interests in mind (Trust)	**1** 9.26(1.26)	**1** 9.59(.89)	**1** 9.48(.92)	**1** 9.22(1.29)
Provider has caring commitment & willing to act on patient’s behalf (Caring)	**2** 9.06(1.39)	**9** 8.81(1.74)	**9** 8.40(1.79)	**9** 8.02(1.83)
Patient & Provider "on the same page" re: patient’s illness & experiences (Shared Mind)	**3** 8.99(1.38)	**4** 9.22(1.43)	**4** 9.00(1.16)	2* 8.83(1.33)
Provider pays full attention to patient during visit (Full Presence)	**4** 8.89(1.55)	**2** 9.38(1.18)	**2** 9.22(1.35)	3* 8.83(1.44)
Provider is open to patient opinions (Shared Deliberation)	**5** 8.83(1.50)	**6** 9.11(1.40)	**7** 8.74(1.41)	**6** 8.48(1.61)
Patient has influence on important issues related to their well-being (Shared Power)	**6** 8.79(1.60)	**8** 8.99(1.48)	**8** 8.63(1.42)	8* 8.41(1.58)
Provider honors patient even if they disagree on important matters (Respect)	**7** 8.28(1.91)	**5** 8.53(1.66)	**5** 8.78(1.35)	**7** 8.47(1.72)
Provider recognizes emotions and provides emotional support (Emotional Support)	**8** 8.17(1.98)	**3** 9.35(1.07)	**3** 9.06(1.26)	**4** 8.60(1.48)
Patient is committed to relationship with provider (Bond)	**9** 8.04(2.20)	**7** 9.08(1.47)	**6** 8.78(1.49)	**5** 8.52(1.68)

Notes: *p < .05; for each * item physicians had significantly lower ratings than the other two groups; APP = advance practice provider (physician assistant or nurse practitioner).

### Provider expectations qualitative results

Recall that there were two questions eliciting expectations from providers; examples of responses for both are shown in Tables 8 and 9. The first question was, “If you could, what would you change about your relationship or interactions with your patients?” We received 312 responses to this question (72% of respondents). Of these, 49% of respondents primarily mentioned that their relationships would be improved if they had more time to spend with patients. Another seven comments mentioned more time as a secondary concern. Comments in the shared deliberation category were mentioned second most, by 18% of respondents. The shared power category received 16% of responses. These comments mostly addressed two different concerns: wishing that patients would take more responsibility for their behavior and health, and that patients would pay less attention to (mis)information on the internet. The remaining comments were spread relatively equally across categories, mostly the TC domains. Although it could be inferred that the large proportion of providers mentioning wishes to spend more time with patients might be experiencing stress related to that, one comment related to caring, made by a physician, summed up what many providers are currently experiencing:

“*I care so much about each and every one of my patients and that takes a lot of energy from me in the process*. *I just wish that I could perhaps care a little bit less and not use up all of my energy on giving to them with nothing left for me at the end of the day*.*”*

**Table 8:** Provider comments on what they would change about patient-provider relationships (n = 312).

What Providers Would Change about Relationships	Category	Number (%) of comments
“Probably what all respondents will say, I wish I had more time to spend with each and every patient!” “That I had more time to really talk to without the time constraints of appointment times.” “Having the time to spend with them…commitment, listening and communication takes time.”	Spend more time with patients	153 (49%)
“Try to make relationships with patients more cooperative. Learn how to motivate people better who don’t want to be motivated, and learn how to talk to patients who don’t see their own agency in their health.” “I would try to have more give and take conversation and be better able to read their response.” “Listen more closely to the patient and be more supportive of their decisions even if not the best decision.”	Shared deliberation	27 (18%)
“I would like for patients to be more willing to take ownership of their health and be willing to do things that are not “easy” or require effort on their part. I would be more apt to go an extra mile for them under these circumstances where I see effort on their part.” “The fact that patients often rely too heavily on outside information such as internet diagnosis and then mistrust” “I would like patients to be more accountable for their health; to keep appointments, and have an idea why they seek care; their medications.”	Shared power	25 (16%)

**Table 9:** Provider comments on what they would change about their workplace (n = 374).

What Providers Would Change about Workplace	Category	Number (%) of comments
“To spend more time ‘present’ with the patient.” “Slow down. Many days we are double and triple booked. This makes it difficult to give each patient the time and attention they deserve.” “I hate having to feel rushed with patients. It’s always go, go, go! And management and surgeons get on you for being too slow. This is how errors happen.”	More time for patients	89 (24%)
“My hospital is not supportive of physicians demonstrating significant compassion.” “To stop feeling like ‘the customer is always right’ attitude by management to get the best HCAHPs scores.” “A more open discussion concerning patient benefit vs company benefit of decisions.” “In an ideal world you could freely express your thoughts and feelings without jeopardizing your job.”	Culture / climate	54 (14%)
“The amount of electronic charting is also a big factor in taking away focused attention from the patient.” “Less reliance on electronics.” “Put all Vocera devices onto a spaceship and send it hurtling into the sun.”	Technology	15 (4%)

The second open-ended question was: “What is one thing you would change about your work environment that would help you have your ideal patient-provider relationship?” Of 374 responses, 24% provided comments related to having more time: some provided very specific, actionable suggestions that would help them have more time, while others simply indicated “more time with patients.” Sub-categories in this theme focused on a few operational topics: the need for less charting/documentation; more staffing/lower nurse-to-patient ratios; fewer interruptions. Frustration with unreliable technology affecting time with patients was expressed frequently. Many respondents (14%) also mentioned topics related to their organization’s culture or climate. Sub-topics in this theme included collaboration, morale, and feeling safe to discuss diverse viewpoints. Other topics included leadership support, and specific operational suggestions (i.e., small group coaching as opposed to large meetings; recognition awards; etc.).

## Discussion

### Key findings

Our quantitative analysis of patient expectations about health care relationships, care provider beliefs about patient expectations, and provider expectations about relationships revealed several key findings. First, and interestingly, respondents ranked “provider always has the patient’s best interest in mind” (trust) as the most important ideal expectation. This means that patients expect that providers will have their best interests in mind, providers know that this is what patients expect, and providers expect to be able to act in the patient’s best interest. While there appears to be concordance on this broader idea, it is not clear how often patients and providers actually agree on what is “best” for the patient. For example, providers could believe they are doing what is best for the patient, and at the same time, the patient could feel not really heard by the provider, or dismissed. Looking at this idea through the provider’s eyes, organizational constraints could prevent doing what they believe is best for the patient. In fact, research on health care worker stress and moral distress has shown that inability to do what is best for patients in accordance with provider professional licenses and training is one of the top factors leading to ethical dilemmas and moral distress among care providers [11, 54].

A second key finding is that providers were remarkably accurate in knowing what patients expect for TCs, especially physicians. The main patient expectation that providers were not accurate about was that providers should have a caring commitment for the patient; patients ranked this second, but providers ranked it from fourth through sixth in their beliefs about what patients expect. The level of concordance between patients and providers suggests that if providers are not able to meet patient expectations in terms of connecting, it could be due to either organizational constraints or barriers related to the patient and their circumstances, not because they do not know what patients expect.

### Key patient results

When comparing the rankings between patients with and without at least one chronic condition, patients with chronic conditions rated being on the same page with providers (shared mind) significantly higher than those without a chronic condition, and they ranked this item as second most important, compared to third for patients with no chronic conditions. As noted above, patients with chronic conditions rated five dimensions significantly higher than did those without chronic conditions (i.e., dimensions of shared mind and deliberation, respect, emotional support, and bond). Walker and Baker noted that as people gain more experience with a particular service or provider, their expectations may become higher [17]. Therefore, it is not too surprising that patients who regularly receive care for chronic conditions might have higher expectations for TCs, especially regarding respect and deliberating about treatment, elements that seem essential for co-production [7, 10]. However, this implies that when such patients access acute care or specialty services they may expect levels of TC they are used to in their primary care setting (predictive expectations), and may therefore be harder to please.

When asked if there was anything else they wanted to say about their provider, patient responses mostly related to dimensions of TCs; however, the largest category of comments fell into technical competence, even though patients were asked to address patient-provider relationships. This is consistent with the general customer expectations literature, in which the dimension of “reliability” (dependability and accuracy of the core service) is generally the most important [41]. Further, the patient trust literature conceptualizes technical quality as an element of trust (i.e., competence) [55]. This is also consistent with our quantitative data that “doing what is best for the patient” (trust) was rated the most important expectation among all the TC dimensions. The explicit examples of technical competence, as indicated in the patient open-ended responses may help us understand what patients mean by “the patient’s best interest.” Similarly, a new theme that emerged and is not explicitly reflected in the dimensions of TCs pertained to patients experiencing their providers as going “above and beyond” in caring for them. This suggests that in these cases providers exceeded patient expectations. Going above and beyond, from the patient’s perspective, may underscore focusing on what is best for the patient: patients seemed to be aware that the patient’s best interest sometimes requires providers to do more than would be typically expected of basic, competent care. Elements mentioned by patients as illustrating going above and beyond included providers making themselves available, and providers doing things they weren’t required to do, such as speaking to administrators on behalf of the patient.

### Key care provider results

As did patients, care providers also ranked having the patient’s best interest in mind as their top expectation for high quality connections with patients; however, providers had some different priorities in terms of ideal expected TC dimensions. Providers ranked having caring commitment to the patient as ninth, while patients ranked it as second. Perhaps providers take caring for granted, given that it is part of their job description. Or, as one care provider commented, perhaps providers try not to care so much; expecting to remain detached in order to reduce risks of burnout. Providers ranked paying full attention to the patient higher than did patients. This makes sense given that paying attention in most cases would enable providers to make accurate diagnoses and treatment plans. The large number of provider comments complaining about distractions, interruptions, and lack of time with the patient underscore this ranking. Surprisingly, providers also ranked recognizing and supporting patient emotions much higher than did patients (patients, 8^th^; providers, 3^rd^ or 4^th^). This suggests that providers prioritize having empathy and compassion. It is possible that this was ranked lower by patients because patients who have never needed emotional support may not expect or value it. Our survey did not measure if patients had ever needed emotional support.

As with the quantitative rankings, providers’ comments indicated good understanding of what patients expect. Most provider comments indicated that patients expect to be able to trust their care providers. A new theme that emerged was related to cultural competence; a small but notable number of providers commented that patients expect providers to understand patient demographic characteristics that could result in patients being marginalized. For this study these comments were coded as “shared mind” because most of the comments pertained to providers “understanding” the impacts of potentially marginalized identities; however, it could be that a new cultural competence dimension should be considered for patient-provider TCs.

Another new theme not reflected in our TC dimensions emerged among providers, which could be related to the highly politicized (in the U.S.) COVID-19 pandemic, or it could be related to a more persistent trend. That is, care providers expressed frustration that patients often express beliefs fueled by misinformation. Given that half of our provider respondents indicated they did not have enough time with patients to establish their ideal TC, the experience of patients bringing misinformation to the encounter, which presumably care providers need to spend time counteracting, likely contributes to a perceived lack of time to build relationships that would help deliver the best care. Although this concern was not expressed by a majority of care providers, it was novel and noteworthy as an aspect of the patient-provider relationship and worth following as misinformation proliferates through social media and other channels.

For provider expectations about relationships, nearly half mentioned that spending more time with patients would enable them to have higher quality connections. This is consistent with Zulman and colleagues’ assertions that providers need to be given the “space” to connect with patients [15]. Presumably, this includes physical and psychological space, but also necessitates the time required to attend to each individual patient and be fully present with them. Providers also made comments about shared deliberation and power being important to them; most shared power comments were focused on patients either doing more to improve their own health, or not bringing (mis)information to the care encounter.

Finally, providers indicated the top thing they would change in their workplaces in order to improve relationships was, again, the ability to spend more time with patients, and they gave actionable suggestions about operational changes that could help them do that. Taken together, these results suggest that not only do providers need to get the service technically correct (correct diagnosis and treatment plan based on what is best for the patient), they also need to consider the relational elements patients expect. Further, it seems that providers generally tend to know this and find it important themselves; but organizations need to be sure the work is designed to enable them to attain this level of co-production. The richness and precision of this study’s data should provide a foundation upon which to draw for further focus on improving value and patient-centeredness.

This study contributes to research on patient-centered care and patient-care provider relationships in three ways. First, prior research has not sufficiently explored the expectations that patients and care providers have about how they will relate with each other during health care encounters. This study demonstrated that patients have ideal expectations for important elements of TCs, and providers generally know what those expectations are. Further, this research importantly adds understanding to what providers ideally want from their connections with patients and show the important ways it differs from what patients want. Second, methodologically, this study offers mirrored survey measures that enable exploring both sides of patient–care provider connections. Third, this study provides quantitative and qualitative evidence regarding the nature and experience of TCs in care encounters as well as the workplace factors that enable or inhibit them, both of which underpin producing truly patient-centered care.

### Future research

Future research should examine the extent to which ideal patient TC expectations are similar for different patient types and those of different demographic groups, such as race/ethnicity and other potentially marginalized identities. The patient sample in this study was located in the U.S., was 85% White, and 93% had health insurance, so there were not enough respondents from potentially marginalized groups to do meaningful comparisons. This should be addressed in future research. Indeed, although research has shown that providing care that is respectful and responsive to individual patient preferences, needs, and values, the definition of patient-centered care, is a universal goal shared across health care systems [56, 57], future research should explicitly examine patient and provider expectations for TCs in numerous cultures. For example, patients (and care providers) in countries with universal access to care may have different expectations for TCs from those in countries without universal access to care. Future research should examine differences in expectations of TCs in relation to differences in access to care as well as the social conditions surrounding care (i.e., social determinants of health). Additionally, differences in preferences and expectations regarding interpersonal interactions may differ across cultures, especially when one party has more expertise and power (provider) than another (patient).

### Practical implications

Our findings have implications for patient-centeredness of care. Our data show that care providers know what patients expect and want on a broad level. However, some challenges arise from the potential diverging TC ideals between patients and providers. Our data found that while patients prioritize a caring commitment on the part of care providers, providers may feel the need to remain emotionally detached in order to prevent emotional exhaustion and burnout. This suggests that organizations need to be better at aligning provider and patient interests—structurally, financially, and in work design and flow. For example, several providers, especially nurses, commented that they are required to carry phones and answer them, even when in the middle of a difficult conversation with a patient. This management requirement essentially prevents providers from being fully present with patients, which could prevent the ability to connect. Research should identify other ways to communicate efficiently and effectively while allowing care providers uninterrupted time with patients.

Through a systematic review of interventions targeted at improving the interpersonal interactions between patients and care providers, Haverfield and colleagues identified educational interventions and tools that positively impact both patient and provider outcomes [28]. For example, tools that aided providers in eliciting patient concerns and stories early in the encounter, led deliberations toward agreements in the care plan, and motivated patients toward self-management, resulted in improved perceptions of the relationship for both patients and providers. Many of these interventions did so without increasing visit times (i.e., cost). Further, some of the interpersonal interventions served to increase provider self-efficacy for connecting, which may enable high quality TCs. Although our data indicated that providers generally know what patients prioritize in terms of connection, future research needs to examine the extent to which providers have self-efficacy for TCs. As such, interventions and tools may improve interpersonal interactions directly and also indirectly by enhancing self-efficacy, which could further improve TCs over time.

### Limitations

As with all research, numerous limitations should be recognized for this study. In general, cross-sectional surveys are limited in that they only capture perceptions at one point in time. Thus, causal relations cannot be inferred. However, this study’s focus was descriptive and did not propose any causal relations. Another limitation of survey designs might be concerns about bias in selection and responding. Although these concerns cannot be completely eliminated, our design and sampling process followed traditional best practices in sampling and survey design. The survey included higher percentages of females versus males than in the general population. While it is typical for randomly sampled survey research to result in more female respondents than males, if females and males in the general population vary systematically on their perceptions about TCs, this could have influenced our findings. One of this study’s limitations may be range restriction with the quantitative items, as most respondents rated most items as relatively highly important. This is not too surprising, as the dimensions examined were already determined in focus groups to be important. It is also not too concerning because there was variability in responding. Finally, our data were collected during the height of the COVID-19 pandemic. Therefore, people who responded to the survey may have been either sicker or more engaged in their care, given that they had received care in the previous year when many patients could not receive any care that was not an emergency. Moreover, during COVID-19 relational concerns might have been especially heightened given the uncertainty of the pandemic and the accompanying social isolation. Studies of TCs during different time periods will help clarify whether this shifts the levels or priorities of the dimensions of TCs. Future research can use causal designs regarding practices to enhance TCs and examine their effects on patient and organizational outcomes.

## Conclusion

This observational study made inroads into unpacking patient and care provider expectations for what comprises a strong TC. It has added richness and precision to our understanding of subtle expectations that may have been overlooked by organizations and care providers. With this foundation, more precise recommendations can be made for using service co-production in the interest of patient-centered care. Organizations must foster the physical and psychological “space” care providers need in order to lead health care service co-production toward true patient-centered care.

## Supporting information

S1 Checklist STROBE statement—Checklist of items that should be included in reports of observational studies. (DOCX)

S1 File Appendix A—study survey instruments. (PDF)

S2 File Patient minimal data set. (XLSX)

S3 File Provider minimal data set. (XLSX)
