# Supplementary material for: What matters to you? An observational field study of patient and care provider expectations for health care relationships
Source: PLoS One. 2024 Jul 2;19(7):e0304854. doi: 10.1371/journal.pone.0304854 (PMC11218989; doi:10.1371/journal.pone.0304854)
Supplement: S1 File — (PDF) [file pone.0304854.s002.pdf]

Appendix A
Study Survey Instruments

Patient Survey

Instructions: We are interested in what makes a good patient-provider relationship, what matters to you as a patient? When we say “provider” that means your IDEAL doctor, nurse, or other type of health care provider.

Please read each of the nine relationship examples below and indicate on the 0-10 scale how important each factor is to you in your ideal patient-provider relationship.

0=Less important 1 2 3 4 5 6 7 8 9 10=More important

1. My provider and I share an understanding of my illness and life experiences; "my provider and I are on the same page."

2. It is possible for me to have influence on important issues regarding my well-being.

3. My provider is open to my opinions and respectful “give and take” during decision making.

4. My provider shows caring commitment to me and the willingness to act on my behalf.

5. My provider pays full attention to me in each moment during a health visit.

6. My provider has my best interests in mind, no matter what.

7. My provider honors me even if he or she disagrees with me on important matters.

8. I feel committed to my relationship with my provider.

9. My provider recognizes when I am feeling difficult emotions and takes actions to support me.

(Open-ended question)
Is there anything else you would like us to know about your relationship with your provider?
(text box)

Care Provider Survey

We are interested in learning about what makes a good patient-provider relationship. **For the following questions please think about what matters in patient-provider relationships from your patients' perspectives. What do you believe matters to your patients?** When we say "provider" that means a patient's main doctor, nurse, or other type of health care provider. There are no "right" or "wrong" answers, please just go with your first response. Note that we are focused only on ***relationships*** right now, not on typical patient experience questions such as timely communication and access to care.

Instructions: Please read each of the nine relationship examples below and indicate on the 0-10 scale **how important you believe each factor is to your PATIENTS** in the patient-provider relationship.

Scale: 0=Less important 1 2 3 4 5 6 7 8 9 10= More important

1. The patient and their provider share an understanding of the patient's illness and life experiences; "my provider and I are on the same page."

2. It is possible for the patient to have influence on important issues pertaining to their well-being.

3. The patient believes their provider is open to their opinions and engages in respectful "give and take" during decision making.

4. The patient believes their provider feels committed to them and a willingness to act on their behalf.

5. The patient believes their provider pays full attention to them in each moment during a health care visit.

6. The patient believes their provider has their best interests in mind, no matter what.

7. The patient believes their provider honors them even if the provider disagrees with them on important matters.

8. The patient feels committed to their relationship with their provider.

9. The patient believes their provider recognizes when they are feeling difficult emotions and takes action to support them.

(Open-ended question)
Are there other key factors in the patient-provider relationship that you believe are important to patients? Again, please focus only on ***relationships.*** (text box)

Next, we want to learn more about patient-provider relationship factors that are important to **YOU**.

Instructions: Please read each of the nine relationship examples below, and think about **YOUR IDEAL patient-provider relationship,** and indicate on the 0-10 scale, **how important it is to YOU**.

0=Less important 1 2 3 4 5 6 7 8 9 10= More important

How important is it to:

1. Share with the patient an understanding of the patient's illness and life experiences; "my patient and I are on the same page."

2. The patient has influence on important issues pertaining to their well-being.

3. Be fully open to the patient's opinions and engage in respectful "give and take" during decision making.

4. Feel commitment to the patient and the willingness to act on their behalf.

5. Always pay full attention to the patient in each moment during a health care visit.

6. Have the patient's best interests in mind, no matter what.

7. Honor the patient even if we disagree on important matters.

8. Being committed to my relationship with the patient.

9. Recognizing when the patient is feeling difficult emotions and taking actions to support them.

(Open ended questions)

If you could, what would you change about your relationships or interactions with your patients? (text box)

What is one thing you would improve about your work environment that would help you have your ideal patient-provider relationship? (text box)
